# Degradation of Carbamazepine from Aqueous Solutions via TiO_2_-Assisted Photo Catalyze

**DOI:** 10.3390/toxics10040168

**Published:** 2022-03-31

**Authors:** Mirela Alina Constantin, Florentina Laura Chiriac, Stefania Gheorghe, Lucian Alexandru Constantin

**Affiliations:** National Research and Development Institute for Industrial Ecology—ECOIND, 57-73 Drumul Podu Dambovitei, Sector 6, 060652 Bucharest, Romania; alina.constantin@incdecoind.ro (M.A.C.); laura.chiriac@incdecoind.ro (F.L.C.); stefania.gheorghe@incdecoind.ro (S.G.)

**Keywords:** AOP, carbamazepine, reactive species, degradation pathway, toxicity

## Abstract

Photocatalytic degradation of carbamazepine (CBZ) from spiked aqueous solutions, via a UV/TiO_2_ system, was investigated, and the optimum photocatalyst type (P25 Degussa) and dose (500 mg/L), as well as irradiation time (45 min), were established. The degradation process kinetics was studied, and a degradation rate constant of 3.14 × 10^−5^ M min^−1^ was calculated for CBZ, using the Langmuir–Hinshelwood equation. Experiments performed in the presence of scavengers showed that the main reactive species involved in the degradation process are holes and free hydroxyl radicals; superoxide radicals also play a role in CBZ degradation. Eight transformation products of CBZ were identified, and a possible degradation pathway, consisting of four routes, was proposed. Toxicity and genotoxicity tests were also performed for both untreated and treated CBZ solutions, proving that the use of a UV/TiO_2_ system represents a suitable treatment approach for aqueous systems with CBZ content.

## 1. Introduction

Pharmaceuticals and personal care products (PPCPs) enter bodies of water in various ways. Although most of them are present in low concentrations, they have complex structures and difficult degradation paths [1,2,3]. Despite the low concentrations, in long-term pollution, PPCPs may be the cause of multiple endocrine diseases, inducing changes in the biochemical functions of aquatic habitats and affecting the biochemical functions of aquatic systems [4].

Carbamazepine CBZ (commercially known as Taver, Tegretol, or Finlepsin) is used in the treatment of cases of epileptic seizure, psychomotor epilepsy, trigeminal neuralgia, pituitary diabetes insipidus, and rebellious hiccups, and it is discharged into the environment in a variety of ways. CBZ absorption in the body is low; only 1 to 2% is excreted in the urine, and 22% reaches the environment through the feces. However, conventional wastewater treatment plants are not capable of efficiently removing PPCPs and their degradation by-products [5,6].

CBZ was found in concentrations of about 0.2 ng/L–11.6 μg/L in water, 0.14–237.1 ng/g dry weight (d.w). in sediment, 1.6 ng/g in fish liver (wet weight—w.w.), 0.88 ng/g in fish muscle (w.w.), and 0–1.9 ng/g in bivalve spp. (w.w.) [7]. According to the globally harmonized system (GSH), the hazard statements attributed to CBZ are H302: harmful if swallowed [warning acute toxicity, oral]; H317: may cause an allergic skin reaction [warning sensitization, skin]; H334: may cause allergy or asthma symptoms, or breathing difficulties, if inhaled [danger sensitization, respiratory] [8]. The CBZ oral LD50 for rats was estimated to be 1957 mg/kg. CBZ overdose may cause various cardiovascular, neurological, respiratory, and urinary symptoms, as well as laboratory abnormalities, including leukocytosis, reduced leucocytes, acetonuria, and glycosuria [9]. The ecotoxicity effects of CBZ were studied in various taxonomic groups, such as *Lumbriculus variegatus* (0.625 and 10 mg/kg (d.w.) for 28 days); *Chironomus riparius* (0.16 to 100 mg/kg d.w.); *Potamopyrgus antipodarum* (28 days, 0.4 to 250 mg/L). In the oligochaete and the snail, no effects were observed. For *C. riparius*, no observed effects were reported at concentrations ranging from 33 to 140 µ/kg d.w., while a 10% effect was reported at concentrations between 70 and 210 µ/kg d.w., based on the carbamazepine concentrations measured in sediments. These results suggest that CBZ may pose a potential threat for the survival of *C. riparius*, and possibly also for other aquatic insect populations in the field [10]. The most sensitive biological system to CBZ was the Vero cell line (monkey kidney cells), followed by *Chlorella vulgaris, Vibrio fischeri, Daphnia magna, Allium cepa*, and RTG-2 cells (salmonid fish cells). EC50 values ranging from 19 µM in Vero cells at 72 h to more than 1200 µM in other systems were obtained. Comparing the concentrations from natural systems and the toxicity quantified in laboratory experiments, CBZ is not expected to produce acute toxic effects in the aquatic biota [11]. A recent study reported the following acute toxic concentrations for CBZ: LC50 (96 h) *Cyprinus carpio* 42.60 mg/L, EC50 (48 h) *Daphnia magna* 21.87 mg/L, and IC50 bacteria 54.21 mg/L. The aquatic risk of CBZ was estimated to be insignificant or low [12]. Based on the CBZ log Kow 2.3–2.77, this compound has low potential for bioaccumulation in aquatic organisms.

Significant concentrations of CBZ and its metabolites were recorded in urban groundwater and Spanish rivers. Moreover, the treatment of CBZ using wastewater treatment plants proved to be inefficient [13].

In Germany, CBZ was detected as human metabolites and transformation products in groundwater, surface waters, and wastewater. Research results proved that these contaminants presented a potential danger to mammals, including humans. A recent research study on CBZ levels, related to the demographic indicators in the groundwater of densely populated areas, showed some worrying statistical results. It appears that correlations exist between the CBZ residuals detected in the groundwater and the aging or social characteristics of a population. The demographic characteristics and drug use in an urban area could influence the quality of potable water sources [14].

Relatively recent studies showed that CBZ is one of the most detected pharmaceutical compounds in Romanian waters, appearing in concentrations varying from 5.4 to 15.1 ng/L [12,15]. This indicates that CBZ is not being efficiently removed by traditional wastewater treatment methods.

Various methods for CBZ removal from wastewater were proposed, including biodegradation, chemical oxidation (presented within Table 1), adsorption, activated sludge processes, and gamma radiation coupled with microbiological treatment, each of them presenting some drawbacks. Advanced oxidation processes (AOPs) were also investigated for CBZ degradation [16], and the preliminary results showed that they can be considered as promising methods for the degradation of recalcitrant pollutants, such as CBZ.

Therefore, in order to surpass the limitations of conventional wastewater treatment processes, the photocatalytic degradation of CBZ, using a UV/TiO_2_ system, was investigated.

## 2. Materials and Methods

### 2.1. Laboratory Set-Up

Photocatalytic treatment was performed using a Heraeus-type UV–Vis (Heraeus, Germany) reactor equipped with an immersed TQ-150-Z3 medium-pressure mercury lamp, with emission spectrum located in the range λ = 320–550 nm; a transparent quartz glass cooling jacket for UV–Vis radiation; a reaction vessel, Vu = 400 cm^3^; a power source: *p* = 150 W, 230 V/50 Hz. The UV lamp’s incident photon flow of 1.15 × 10^−6^ einstein^−1^ was determined using the ferrioxalate actinometrical method [19].

All samples were continuously homogenized during UV irradiation using a VELP SCIENTIFICA magnetic stirrer, with an adjustable speed; *p* = 40 W, 230 V/50 Hz.

All experiments were performed at room temperature 23 ± 2 °C and pH = 6. All photocatalytic experiments were duplicated, and average values were considered.

### 2.2. Reagents

The following reagents were used within the photocatalytic degradation tests: carbamazepine 98% (Acros Organics, purchased from Thermo Fisher, Waltham, MA, USA), titanium dioxide P25 Degussa (purchased from Sigma Aldrich, Darmstadt, Germany), titanium dioxide Rovis Optics anatase (purchased from Sigma Aldrich, Darmstadt, Germany), titanium dioxide Merck (purchased from Merck Romania, Bucharest, Romania), titanium dioxide Kurt Lesker (purchased from Kurt J. Lesker, Dresden, Germany), and titanium dioxide Umicore (purchased from Umicore, Essen, Germany). For analytical measurements, HPLC-grade acetonitrile was purchased from Sigma Aldrich, and ultrapure water was obtained using Millipore Milli-Q (Millipore purchased from Merck Romania, Bucharest, Romania) equipment.

### 2.3. Analytical Methods

The carbamazepine concentration was determined using an Agilent 1260 liquid chromatograph, coupled with an Agilent 6410B triple quadrupole mass spectrometer with an electrospray ionization source (ESI). Carbamazepine was eluted on a chromatographic column with a hydrophobic stationary phase, type C18 (Synergy Fusion-RP, 150 × 2.0 mm, 4.0 μm, Phenomenex). To monitor the carbamazepine concentration and the formation of unknown degradation products, MS/MS detection was performed in SCAN mode. The mobile phase, consisting of ultrapure water (A) and acetonitrile (B), was eluted in the isocratic mode, in a ratio of 70% (A)/ 30% (B), with a flow rate of 0.2 mL/minute. The temperature of the chromatographic column was maintained at 25 °C, and the injection volume was 10 μL. To identify degradation products, full-scan chromatograms were recorded, using both electrospray ionization sources in positive mode. The scan range was between 80 and 300 Da. The values of the ionization source parameters were capillary voltage of 4000 V, drying gas temperature of 300 °C, drying gas flow of 8 L/min, and nebulizer pressure of 40 psi.

The analytical method was developed and validated in the present study for the detection and quantification of carbamazepine in aqueous solutions. All details regarding the method validation parameter values are given in the Supplementary Materials as Method S1.

### 2.4. Toxicity and Genotoxicity Tests

Two acute tests with the following freshwater organisms were performed: *Daphnia magna* (planktonic crustacean) [20] and *Pseudokirchneriella subcapitata* (green algae) [21]. The tests were miniaturized using Daphtoxkit F Magna and Algaltoxkit F microbiotests provided by Microbiotest Belgium. The endpoints of tests were mortality percentages at 24 h and 48 h in the case of *Daphnia*, and growth rate inhibition at 72 h in the case of algae. In order to assess CBZ genotoxicity, an EBPI SOS-Chromotest (Environmental Bio-Detection Products), with an engineered *Escherichia coli* strain, was applied. The test is based on bacteria’s capacity to detect the genotoxic activity of chemicals at the genetic material (DNA) level by defense-specific enzymatic protein expression. In this case, the target gene encodes β-galactosidase (β-gal). Bacteria viability checking, based on the presence of alkaline phosphatase, was performed using a blue chromogen compound (a yellow to green color indicates bacteria viability) [22,23].

## 3. Results

A relatively recent study, focusing on the use of five commercial TiO_2_ suspended photocatalysts (different from those used in our study) for CBZ degradation, used CBZ concentrations in the range of 5–20 mg/L [24]. In order to assure comparison with this specific study, an initial concentration [CBZ]_0_ = 8.75 mg/L = 3.71 × 10^−5^ M was chosen for our experiments, even though this concentration is higher than those used in real cases. In the first stage, the influence of photocatalyst type vs. CBZ degradation efficiency was assessed.

### 3.1. Influence of the Photocatalyst Type

In order to investigate the influence of the photocatalyst type, photo-oxidation experiments were performed, using the following five types of photocatalysts: P25 Degussa, Rovis Optics anatase, Merck, Kurt Lesker, and Umicore.

Prior to irradiation, the photocatalyst dose was added to the samples, and the obtained suspensions were bubbled with air (50 L/h) for 30 min in the dark, in order to avoid a hole–electron recombination process.

The samples were continuously homogenized within the reactor using a magnetic stirrer.

The obtained experimental data are presented in Table 2.

### 3.2. Influence of Photocatalyst Dose and Irradiation Time

**Figure 1:**
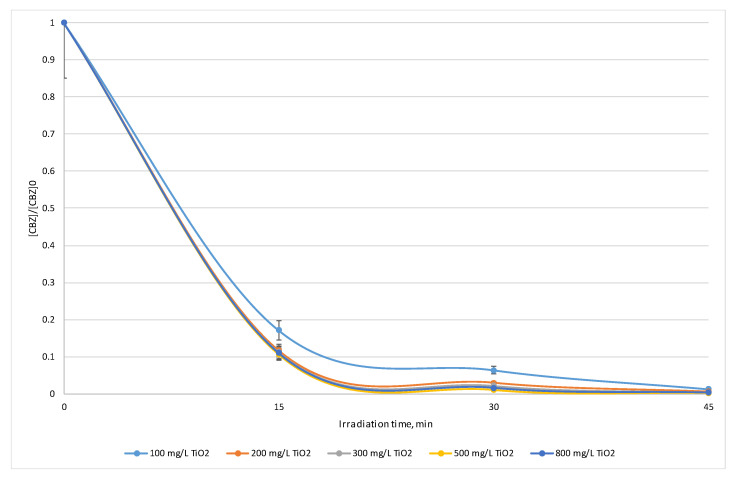
Normalized CBZ concentration for various catalyst (P25 Degussa) doses vs. irradiation time.

In order to establish the optimum photocatalyst dose, CBZ degradation experiments were performed for the same initial concentration of the target pollutant, [CBZ]_0_ = 8.75 mg/L = 3.71 × 10^−5^ M, while varying the irradiation time between 15 and 45 min, and the photocatalyst dose between 100 and 800 mg/L (P25 Degussa). The obtained experimental results are shown in Table S1. The obtained results are also supported by the normalized CBZ concentrations versus time profiles, which are shown in Figure 1.

### 3.3. Effect of Scavengers’ Presence

In order to investigate the main species involved in CBZ degradation, the experiments were performed using the same spiked solution, with an initial concentration [CBZ]_0_ = 8.75 mg/L = 3.71 × 10^−5^ M, photocatalyst dose = 500 mg/L, irradiation time = 45 min, in the presence of the following scavengers:

- NaF (sodium fluoride) (50:1)—for blocking hydroxyl radicals from the photocatalyst particle’s surface;
- i-PrOH (isopropanol) (100:1)—for blocking free hydroxyl radicals HO;
- EDTA (disodium ethylenediaminetetracetate) (100:1)—for blocking holes h+;
- BQ (1,4-benzoquinone) (45:1)—for blocking superoxide radicals O_2_.

**Figure 2:**
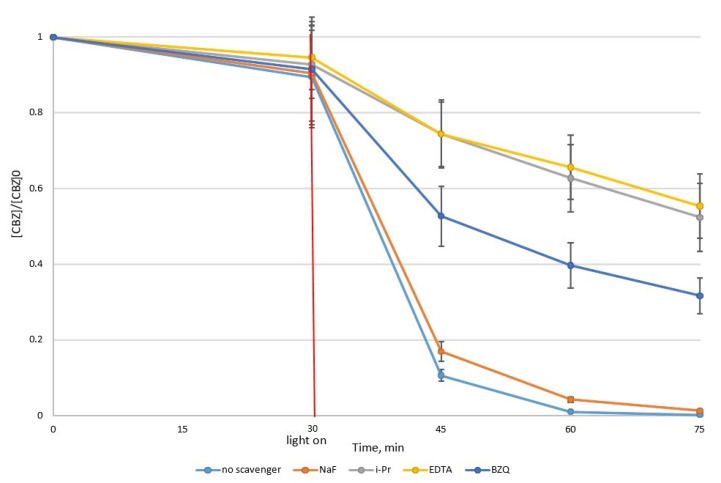
Normalized CBZ concentration vs. irradiation time in the presence and absence of various scavengers.

The results obtained in the scavenger’s presence were compared with those obtained in the scavenger’s absence, and these are presented in Figure 2.

### 3.4. Identification of CBZ Degradation By-Products

During the degradation experiments, it was observed that the peak area of CBZ decrease coincided with the appearance of new, unknown peaks, confirming the efficient degradation of CBZ and the formation of transformation products. Being more polar, the detected transformation products (TPs) were eluted from the LC-MS chromatographic column at lower retention times compared to CBZ, while [M+H]^+^ was determined using the MS detector. Following LC-MS/MS analysis, eight TPs were observed and identified. Based on the molecular ion masses resulting from the MS2 fragmentation patterns, possible chemical structures were proposed for each transformation product. The [M+H]^+^ molecular ion peaks, retention times, molecular structures, and chemical formulas for both CBZ and TPs are given in Table 3.

### 3.5. Toxicity and Genotoxicity Tests

In order to assess the toxicity of both the initial CBZ solution [CBZ]_0_ = 8.75 mg/L = 3.71 × 10^−5^ M and the UV/TiO_2_-treated solutions, using optimum operational parameters, the toxicity effects upon *Daphnia magna* planktonic crustaceans and *Pseudokirchneriella subcapitata* green algae, and the genotoxicity effects upon *Escherichia coli* bacteria, were investigated, and the results are presented in Table 4.

## 4. Discussion

### 4.1. Optimum Photocatalyst Type

Among the commercial photocatalysts, P25 Degussa proved to be the most efficient in the photocatalytic degradation of CBZ, achieving a degradation efficiency of 93.60% after 30 min of irradiation with k = 1.53 × 10^−3^ s^−1^. Good degradation efficiencies were also achieved for Rovis Optics anatase (88.0%) and Merck photocatalysts (85.03%), while the degradation efficiency was very low for the other two tested photocatalysts. Therefore, P25 Degussa was selected to be used in further experiments.

### 4.2. Optimum Photocatalyst Dose

Although good degradation efficiencies were obtained under all the experimental conditions tested (>80% in all cases), the experimental results proved that the optimum photocatalyst dose for CBZ degradation is [TiO_2_] = 500 mg/L, which assures a 99.77% degradation efficiency (CBZ residual concentration = 0.02 mg/L = 8.47 × 10^−8^ M) for an irradiation time of 45 min.

The increase in photocatalyst dose led to an increase in CBZ degradation efficiency for [TiO_2_] doses, by up to 500 mg/L (due to the increase in active sites), followed by a decrease in CBZ degradation efficiency for [TiO_2_] doses higher than 500 mg/L. This can be explained by the negative influences of light scattering processes and the photocatalyst agglomeration tendency.

On the other hand, as expected, the increase in irradiation time proved to have a positive impact upon CBZ degradation efficiency.

In respect to the obtained experimental results, the optimum CBZ degradation parameters were set up as a photocatalyst dose of 500 mg/L and an irradiation time of 45 min.

### 4.3. Kinetics

Linearization of the CBZ degradation equation, using a pseudo-first-order kinetic (Figure S1), allows the calculation of the degradation rate constant (0.1411 min^−1^, which was similar to other results reported in the literature [25]).

The Langmuir–Hinshelwood equation is widely used to describe the kinetics of UV/TiO_2_ processes. The model was also applied for organic compound degradation in aqueous suspensions [26]. The target pollutant’s degradation rate is described by the following equation:

(1) $$r_{0}=-\frac{d\left[\mathrm{CBZ}\right]}{\mathrm{dt}}=\frac{k_{r}K_{\mathrm{ads}}{\left[\mathrm{CBZ}\right]}_{0}}{1+K_{\mathrm{ads}}{\left[\mathrm{CBZ}\right]}_{0}}$$

where:

r_o_—initial degradation rate of CBZ;

[CBZ]_0_—initial CBZ concentration;

[CBZ]—CBZ concentration at a given t time;

K_ads_—equilibrium constant of CBZ adsorption/desorption on TiO_2_ particles;

k_r_—CBZ degradation rate constant;

t—irradiation time.

Equation (1) can be rearranged as a linear equation, as follows:

(2) $$\frac{1}{r_{0}}=\frac{1}{k_{r}}+\frac{1}{k_{r}K_{\mathrm{ads}}{\left[\mathrm{CBZ}\right]}_{0}}$$

The degradation rate constant k_r_ and equilibrium constant of CBZ adsorption/desorption of photocatalyst particles K_ads_ can be calculated from the Equation (2) graphic plot (Figure S2), where 1/k_r_ represents the intercept and 1/k_r_K_ads_ represents the slope of the graphical plot.

The initial pollutant degradation rate r_0_ (M min^−1^) was calculated from the experimental data after 30 min of irradiation.

In order to plot Equation (2), the initial concentration of CBZ was varied between 1.54 and 8.75 mg/L. From the graph’s slope and intercept, the following values were calculated: k_r_ = 3.14 × 10^−5^ M min^−1^ and Kads = 1062 M^−1^. As the initial CBZ concentration increased (from 1.54 mg/L to 8.75 mg/L), the apparent reaction rate constant decreased (from 0.167 min^−1^ to 0.152 min^−1^). It should be noted that the results follow the same trend recorded by another study [24] that used a P90 photocatalyst and a CBZ concentration range of 5–20 mg/L.

The degradation of CBZ obeys the Langmuir–Hinshelwood model, and the obtained kinetic data suggest that the hydroxyl radicals absorbed on the catalyst surface play a minor role in CBZ degradation.

### 4.4. Main Reactive Species

The presence of NaF proved to have a minimal influence upon CBZ degradation, resulting in a CBZ degradation efficiency of 98.74%, compared with 99.77% obtained in the absence of a scavenger. This behavior suggests minimal involvement of hydroxyl radicals from the photocatalyst surface within CBZ photocatalytic degradation. This is also sustained by the low value of the adsorption/desorption equilibrium constant obtained using the Langmuir–Hinshelwood equation.

Holes and free hydroxyl radicals represent the main active species within CBZ photocatalytic degradation; the presence of EDTA and i-PrOH led to the highest decreases in CBZ degradation efficiency, of 44.69% and 47.66%, respectively.

Superoxide radicals can play a role within CBZ photocatalytic degradation, and the presence of BQ led to a decrease in the CBZ degradation efficiency, to 68.34%.

### 4.5. Possible Degradation Pathway

**Figure 3:**
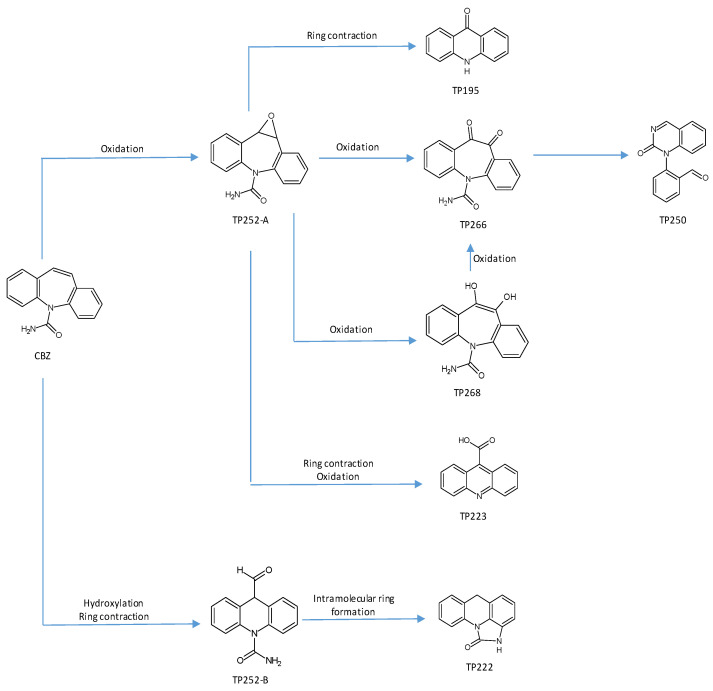
Proposed degradation pathway.

Based on the identified intermediates, their evolution during CBZ degradation (Figure S3), and the recent information available in the literature [25,26], possible pathways for CBZ’s degradation, via TiO_2_-assisted photocatalysis, have been proposed (Figure 3).

The first possible degradation route consists of successive CBZ oxidations, with the formation of TP252-A, followed by ring contraction (from seven to six), with TP195 formation.

The second possible degradation route consists of successive CBZ oxidations, with the formation of TP252-A, which is further transformed into TP250, through scindation of the initial hepta-heterocycle, followed by intramolecular cyclization of a new six-atom heterocycle.

It should be mentioned that those two possible CBZ degradation routes have been confirmed by other recent information reported within the literature for CBZ degradation via titanium dioxide-assisted photocatalysis or other advanced oxidation processes [25,27,28,29,30,31]. Intermediates, such as hydroxyl-carbamazepine TP252-B or acridone TP195, reported in other studies on CBZ degradation, assisted by TiO_2_ [24,30], were also identified in our study.

A third proposed route consists of CBZ successive oxidations to TP268, followed by its transformation into TP266 and further transformation into TP250, through ring contraction and oxidation (successive reactions of heterocycle scindation and intramolecular re-cyclization).

It should be said that successive oxidation of CBZ to TP268 was also reported within other studies from the literature, but for CBZ photolysis, rather than for photocatalysis [25,26].

The fourth proposed route consists of CBZ transformation through hydroxylation and ring contraction into TP252-B, followed by further transformation into TP222 via intramolecular ring formation and aldehyde group elimination.

This route was also reported within the literature, but for CBZ degradation via photo-electro-catalysis, rather than for photocatalysis [25,26].

### 4.6. Toxicity and Genotoxicity

In the case of *Daphnia*, an acute toxic effect of 95–100% for the initial CBZ solution was recorded after 24 h and 48 h, respectively. Toxic effects were also registered for irradiated samples after both 24 h and 48 h of exposure. However, the toxic effect decreased in comparison with the initial CBZ solution, from 95% to 10%, after 24 h, and from 100% to 70% after 48 h of exposure. This finding can be explained by the fact that the tested solutions did not have an immediate toxic effect, but, rather, a chronic effect. Another possible explanation could be the formation of toxic compounds during CBZ photocatalytic degradation. However, the acute toxicity of the irradiated samples was lower compared with that of the initial CBZ solution. The test showed that the toxicity effects decrease with the decrease in CBZ concentration in the tested solutions, and with the increase in UV irradiation time.

Neither the initial CBZ solution nor the irradiated samples determined toxic effects upon algae (they did not affect algal growth). On the contrary, growth stimulation was observed, especially for samples treated for a longer irradiation time. The effects registered after 48 h and 72 h for the tested samples indicate adaptation/tolerance to the toxic compound, more accentuated for those treated for a longer irradiation time (with a lower CBZ concentration).

The level of genotoxicity was measured by calculating the induction factor (IF). The analyzed samples presented an IF < 1.5 (1.20–1.28, respectively), indicating non-genotoxicity effects upon *Escherichia coli* bacteria. The positive control presented an induction factor of IF ≥ 2 at most 4NQO concentrations, indicating the test’s validity. The survival rates of bacteria were higher than 98% for all the tested samples, suggesting that there were no cytotoxic effects of untreated or treated solutions of CBZ.

The results are in accordance with other studies from the literature, which proved that CBZ presents relatively limited acute ecotoxicity [11,31,32,33,34,35,36].

## 5. Conclusions

The photocatalytic degradation of CBZ was studied using a spiked CBZ solution, with an initial concentration [CBZ]_0_ = 8.75 mg/L = 3.71 × 10^−5^ M. Five types of commercial titanium dioxide catalysts were tested, and Degussa P25 was selected as the optimum. The influence of photocatalyst dose and irradiation time was investigated, and an optimum titanium dioxide dose of 500 mg/L and an irradiation time of 45 min were found to allow a CBZ degradation efficiency of 99.77%, with an apparent CBZ degradation rate constant of 0.1411 min^−1^. Appliance of the Langmuir–Hinshelwood model allows for the calculation of the CBZ degradation rate constant as k_r_ = 3,14 × 10^−5^ M min^−1^, and the equilibrium constant of CBZ adsorption/desorption of the photocatalyst surface as K_ads_ = 1062 M^−1^. The tests performed in the presence of various scavengers showed that the main CBZ degradation routes are those through holes and free hydroxyl radicals; superoxide radicals also play a role in CBZ degradation. Hydroxyl radicals from the catalyst surface play a minor role in CBZ degradation in the above-mentioned experimental conditions. Eight CBZ transformation products were identified and four possible routes for CBZ degradation were proposed, the majority of which consist of successive oxidation reactions. From the four proposed degradation routes, two were not found to be reported within the literature for the photocatalytic degradation of CBZ, but only for photolysis and photo-electro-catalysis. Toxicity assays proved that both the CBZ initial solution and irradiated samples presented acute toxicity upon *Daphnia magna* planktonic crustaceans, with treated samples being less toxic compared with the initial untreated CBZ solution. Both the initial untreated CBZ solution and irradiation samples present no toxic effects upon *Pseudokirchneriella subcapitata* green algae and no genotoxic effects upon *Escherichia coli*.

## Figures and Tables

**Table 1:** CBZ efficiency removal via conventional processes.

Process	[CBZ]	Removal Efficiency	Reference
Biodegradation with fungi	4 μg/L–9 mg/L	40–90%	[17]
Biodegradation with bacteria	4 μg/L–9.5 mg/L	15–60%	[17]
Chemical oxidation with Fe (II) activated persulfate	0.025 mM	78%	[18]

**Table 2:** CBZ degradation efficiencies obtained using various catalyst types, [CBZ]_0_ = 8.75 mg/L = 3.71 × 10^−5^ M, irradiation time = 30 min, photocatalyst dose = 100 mg/L.

Photocatalyst Type	[CBZ], mg/L	Efficiency, %	Apparent First-Order Reaction Rate Constant (Mean Value) k, s^−1^
P25 Degussa	0.56 ± 0.09	93.60 ± 1.04	1.53 × 10^−3^
Rovis Optics anatase	1.05 ± 0.17	88.00 ± 2.81	1.18 × 10^−3^
Merck	1.31 ± 0.20	85.03 ± 2.49	1.06 × 10^−3^
Kurt Lesker	7.60 ± 0.40	13.14 ± 4.57	7.83 × 10^−5^
Umicore	8.01 ± 0.40	8.46 ± 4.68	4.91 × 10^−5^

**Table 3:** Identified CBZ transformation products.

No	Structure Name	Retention Time, min	Molecular Ion, [M + H]^+^	Molecular Formula	Chemical Structure
1	CBZ	12.1	237	C_15_H_12_N_2_O	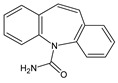
2	TP268	3.15	269	C_15_H_12_N_2_O_3_	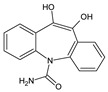
3	TP266	3.37	267	C_15_H_10_N_2_O_3_	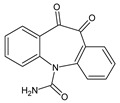
4	TP195	3.63	196	C_13_H_9_NO	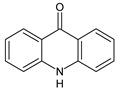
5	TP222	3.66	223	C_14_H_10_N_2_O	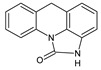
6	TP252-A	4.42	253	C_15_H_12_N_2_O_2_	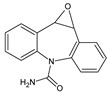
7	TP252-B	4.79	253	C_15_H_12_N_2_O_2_	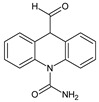
8	TP250	5.25	251	C_15_H_10_N_2_O_2_	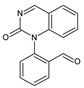
9	TP223	5.32	224	C_14_H_9_NO_2_	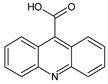

**Table 4:** Acute toxicity and genotoxic effects of CBZ solutions *.

	*Daphnia magna* (Mortality %)		*Pseudokirchneriella subcapitata* (Algae Growth Inhibition Rate %)			*Escherichia coli* (Genotoxicity)	
Sample	24 h	48 h	24 h	48 h	72 h	Survival Rate, %	Genotoxicity IF
Untreated [CBZ] = 8.75 mg/L	95 ± 19	100 ± 20	−48.18 ± (−14.4)	1.77 ± 0.53	3.53 ± 1.05	107.34 ± 21.4	1.23 ± 0.23 (NO)
15 min irradiation [CBZ] = 0.93 mg/L	45 ± 9	95 ± 19	−46.04 ± (−13.81)	16.17 ± 4.85	8.40 ± 2.52	104.81 ± 20.96	1.20 ± 0.23 (NO)
30 min irradiation [CBZ] = 0.09 mg/L	25 ± 5	75 ± 15	77.91 ± 23.37	−11.71 ± (−3.51)	−2.22 ± (−0.66)	N/A	N/A
45 min irradiation [CBZ] = 0.02 mg/L	10 ± 2	70 ± 14	−211.69 ± (−63.3)	−9.39 ± (−2.81)	−2.62 ± (−0.78)	98.27 ± 19.65	1.28 ± 0.20 (NO)
Controls No CBZ	0	0	0	0	0	NC 96.88 PC 100%	1.05 ± 0.21 (NO) 3.68 ± 0.77 (YES)

* Cumulative final effect (24 h and 48 h); “-“ algae growth rate stimulation; NO/YES—detection of genotoxic effects (according to test protocol and induction factor IF < 1.5, indicating no genotoxicity, NC—negative control, PC—positive control or genotoxicity (with 4-nitroquinoline-1-oxide (4NQO)). Results are expressed as averages of three replicates (*n* = 3) ± standard deviation (SD).

## Data Availability

Data are contained within the article and Supplementary Materials.

## Supplementary Materials

The following supporting information can be downloaded at https://www.mdpi.com/article/10.3390/toxics10040168/s1: Figure S1: linearization of CBZ degradation in UV/TiO_2_ system by pseudo-first-order kinetics, [TiO_2_] = 500 mg/L, [CBZ]_0_ = 8.75 mg/L = 3.71 × 10^−5^ M, irradiation time = 45 min; Figure S2: linearization of Langmuir–Hinshelwood equation for CBZ degradation via UV/TiO_2_ system; Figure S3: profiles of TPs evolution during CBZ degradation process, A—TP peak area, A0—CBZ initial peak area; Table S1: CBZ degradation efficiencies vs. photocatalyst dose and irradiation time, [CBZ]_0_ = 8.75 mg/L = 3.71 × 10^−5^ M, photocatalyst dose = 100–800 mg/L, irradiation time = 15–45 min; Method S1: CBZ determination method validation.

## References

[1] Chen H., Gu X., Zeng Q., Mao Z. (2019). Acute and chronic toxicity of carbamazepine on the release of chitobiase, molting, and reproduction in Daphnia similis. Int. J. Environ. Res. Public Health.

[2] Yang Y.Y., Zhao J.-L., Liu Y.-S., Liu W.-R., Zhang Q.-Q., Yao L., Hu L.-X., Zhang J.-N., Jiang Y.-X., Ying G.-G. (2018). Pharmaceuticals and personal care products (PPCPs) and artificial sweeteners (ASs) in surface and ground waters and their application as indication of wastewater contamination. Sci. Total Environ..

[3] Constantin L.A., Constantin M.A., Nitoi I., Chiriac F.L., Galaon T., Cristea N.I. (2018). Possible pathway for ifosfamide degradation via Fe-TiO_2_ assisted photo catalysis. Rev. Chim.–Bucharest.

[4] Tran R.H., Reinhard M., Gin K.Y.-H. (2018). Occurrence and fate of emerging contaminants in municipal wastewater treatment plants from different geographical regions–a review. Water Res..

[5] Tarpani R.R.Z., Azapagic A. (2018). A methodology for estimating concentrations of pharmaceuticals and personal care products (PPCPs) in wastewater treatment plants and in freshwaters. Sci. Total Environ..

[6] Zhang Y., Geiben S.-U., Gal C. (2008). Carbamazepine and diclofenac: Removal in wastewater treatment plants and occurrence in water bodies. Chemosphere.

[7] Batucan N.S.P., Tremblay L.A., Northcott G.L., Matthaei C.D. (2022). Medicating the environment? A critical review on the risks of carbamazepine, diclofenac and ibuprofen to aquatic organisms. Environ. Adv..

[8] Compound Summary Carbamazepine. https://pubchem.ncbi.nlm.nih.gov/compound/Carbamazepine#section=Hazards-Identification.

[9] Carbamazepine. https://go.drugbank.com/drugs/DB00564.

[10] Oetken M., Nentwig G., Löffler D., Ternes T., Oehlmann J. (2005). Effects of pharmaceuticals on aquatic invertebrates. Part I. The antiepileptic drug carbamazepine. Arch. Environ. Contam Toxicol..

[11] Jos A., Repetto G., Rios J.C., Hazen M.J., Molero M., del Peso A., Salguero M., Fernandez-Freire P., Perez-Martin J.M., Camean A. (2003). Ecotoxicological evaluation of carbamazepine using six different model systems with eighteen endpoints. Toxicol. In Vitro.

[12] Gheorghe S., Petre J., Lucaciu I., Stoica C., Nita-Lazar M. (2016). Risk screening of pharmaceutical compounds in Romanian aquatic environment. Environ. Monit. Assess..

[13] Xie Z., Lu G., Yan Z., Liu J., Wang P., Wang Y. (2017). Bioaccumulation and trophic transfer of pharmaceuticals in food webs from a large freshwater lake. Environ. Pollut..

[14] Ebrahimzadeh S., Castiglioni S., Riva F., Zuccato E., Azzellino A. (2021). Carbamazepine Levels Related to the Demographic Indicators in Groundwater of Densely Populated Area. Water.

[15] Petre J., Galaon T., Vasile I.I., Vasile G.G., Stanescu E., Pascu L.F., Simion M., Cruceru L. (2016). Simultaneous analysis of selected dissolved pharmaceuticals in the water of the Danube river and its three major tributaries in Romania. Rev. Chim.-Bucharest.

[16] Wang S., Wang J. (2018). Degradation of carbamazepine by radiation-induced activation of peroxymonosulfate. Chem. Eng. J..

[17] Nasir N.M., Talib S.A., Hashim S.N., Tay C.C. (2017). Biodegradation of carbamazepine using fungi and bacteria. J. Fundam. Appl. Sci..

[18] Rao Y.F., Qu L., Yang H., Chu W. (2014). Degradation of carbamazepine by Fe (II) activated persulfate process. J. Hazard. Mater..

[19] Calvert J.G., Pitts J.N. (1966). Photochemistry.

[20] Technical Committee ISO/TC 147/SC 5 Biological methods (2012). Water Quality–Determination of the Inhibition of the Mobility of *Daphnia Magna* Straus (Cladocera, Crustacea)–Acute Toxicity Test.

[21] Technical Committee ISO/TC 147/SC 5 Biological methods (2012). Water Quality–Fresh Water Algal Growth Inhibition Test with Unicellular Green Algae.

[22] EBPI Specializing in Biomolecular Testing Kits for Toxicity, Mutagenicity, and Genotoxicity; Environmental Bio-Detection Products Inc. Ontario, Canada. https://www.biotoxicity.com/index.php/ebpi-toxicity-tests/sos-genotoxicity-tests/60-ebpi-toxicity-tests/201-sos-genotoxicity-tests.

[23] Kocak E. (2015). Investigation of potential genotoxic activity using the SOS-Chromotest for real paracetamol wastewater and the wastewater treated by the Fenton process. J. Environ. Health Sci. Engineer.

[24] Carabin A., Drogui P., Robert D. (2015). Photo-degradation of carbamazepine using TiO_2_ suspended photocataysts. J. Taiwan Inst. Chem. Eng..

[25] Franz S., Falletta E., Arab H., Murgolo S., Bestetti M., Mascolo G. (2020). Degradation of carbamazepine by photo(electro)catalysis on nanostructured TiO_2_ meshes: Transformation products and reaction pathways. Catalysts.

[26] Murgolo S., Franz S., Arab H., Bestetti M., Falletta E., Mascolo G. (2019). Degradation of emerging organic pollutants in wastewater effluents by electrochemical photocatalysis on nanostructured TiO_2_ meshes. Water Res..

[27] Xu L., Niu J., Xie H., Ma X., Zhu Y., Crittenden J. (2021). Effective degradation of aqueous carbamazepine on a novel blue-colored TiO_2_ nanotube arrays membrane filter anode. J. Hazard. Mater..

[28] Yang L., Hao X., Yu D., Zhou P., Peng Y., Jia Y., Zhao C., He J., Zhan C., Lai B. (2021). High visible-light catalytic activity of Bis-PDI-T@TiO_2_ for activating persulfate toward efficient degradation of carbamazepine. Sep. Purif. Technol..

[29] Yang L., Jia Y., Peng Y., Zhou P., Yu D., Zhao C., He J., Zhan C., Lai B. (2021). Visible-light induced activation of persulfate by self-assembled EHPDI/TiO_2_ photocatalyst toward efficient degradation of carbamazepine. Sci. Total Environ..

[30] Dudziak S., Bielan Z., Kubica P., Zielinska-Jurek A. (2021). Optimization of carbamazepine photodegradation on defective TiO_2_–based magnetic photocatalyst. J. Environ. Chem. Eng..

[31] El Mragui A., Logvina Y., da Silva L.P., Zegaoui O., da Silva J.C.G.E. (2019). Synthesis of Fe–and Co- doped TiO2 with improved photocatalytic activity under visible irradiation toward carbamazepine degradation. Materials.

[32] Ferrari B., Paxeus N., Lo Giudice R., Pollio A., Garric J. (2003). Ecotoxicological impact of pharmaceuticals found in treated wastewaters: Study of carbamazepine, clofibric acid, and diclofenac. Ecotox. Environ. Safe..

[33] Jones O.A.H., Voulvoulis N., Lester J.N. (2002). Aquatic environmental assessment of the top 25 English prescription pharmaceuticals. Water Res..

[34] Laville N., Ait-Aissa S., Gomez E., Casellas C., Porcher J.M. (2004). Effects of human pharmaceuticals on cytotoxicity, EROD activity and ROS production in fish hepatocytes. Toxicology.

[35] Donner E., Kosjek T., Qualmann S., Kusk K.O., Michael Revitt D., Ledin A., Andersen H.R. (2013). Ecotoxicity of carbamazepine and its UV photolysis transformation products. Sci. Total Environ..

[36] Pan F., Ji H., Du P., Huang T., Wang C., Liu W. (2021). Insights into catalytic activation of peroxymonosulfate for carbamazepine degradation by MnO_2_ nanoparticles in-situ anchored titanate nanotube: Mechanism, ecotoxicity and DFT study. J. Hazard. Mater..

