# Supplementary material for: Degradation of Carbamazepine from Aqueous Solutions via TiO2-Assisted Photo Catalyze
Source: Toxics. 2022 Mar 31;10(4):168. doi: 10.3390/toxics10040168 (PMC9032724; doi:10.3390/toxics10040168)
Supplement: Supplementary file 1 [file toxics-10-00168-s001.zip › toxics-1640486-supplementary.pdf]

toxics MDPI

Supplementary Materials: Degradation of Carbamazepine from Aqueous Solutions via TiO_2-Assisted Photo Catalyze

Mirela Alina Constantin, Florentina Laura Chiriac, Stefania Gheorghe and Lucian Alexandru Constantin

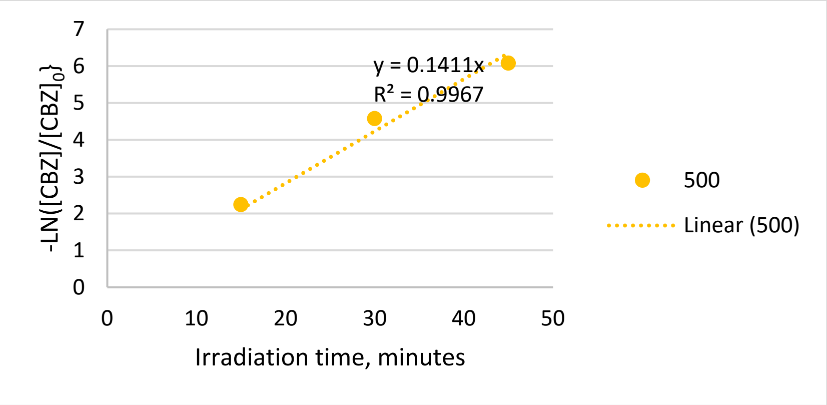

Figure S1. Linearization of CBZ degradation in UV/TiO2 system by pseudo-first order kinetic, [TiO2] = 500 mg/L, [CBZ]0 = 8.75 mg/L = 3.71×10^{-5} M, irradiation time = 45 min.

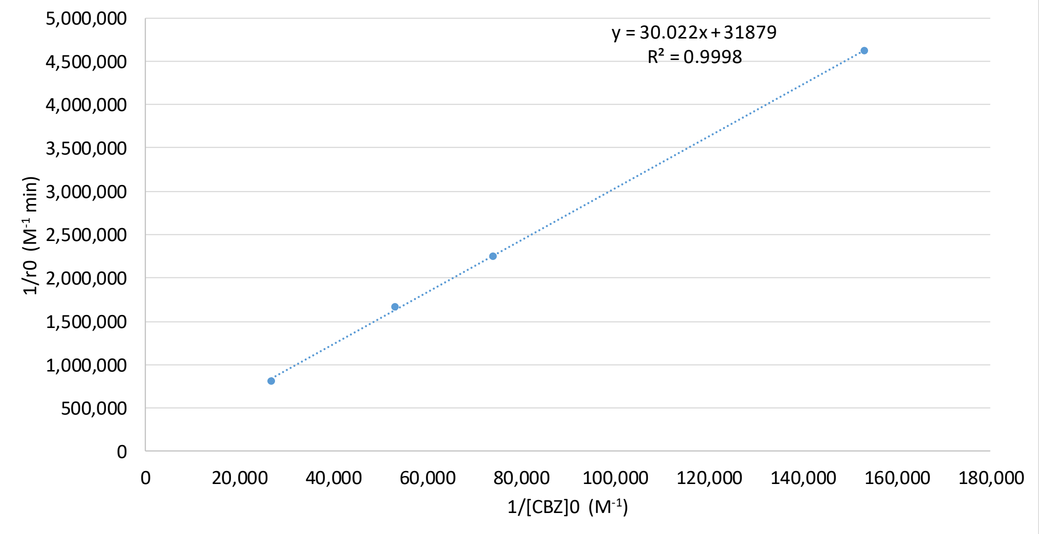

Figure S2. Linearization of Langmuir-Hinshelwood equation for CBZ degradation via UV/TiO_2 system.

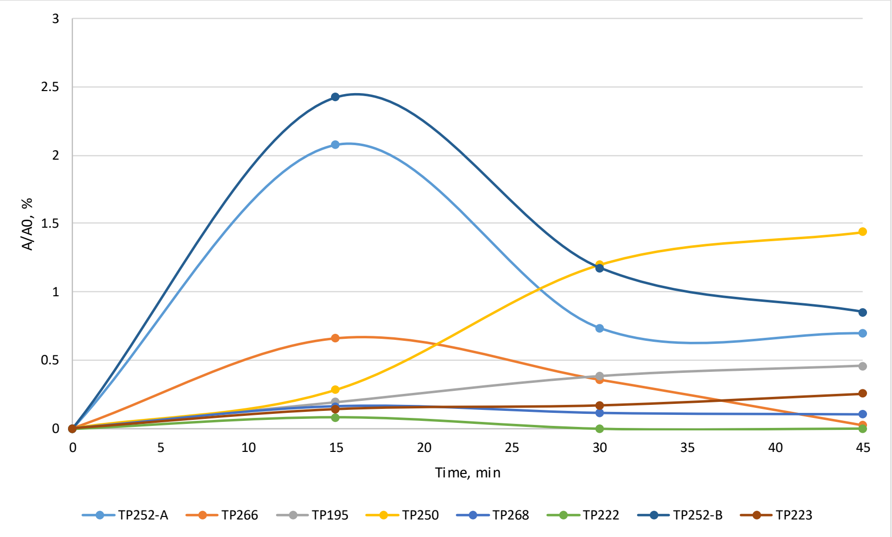

Figure S3. Profiles of TPs evolution during CBZ degradation process, A-TP peak area, A0 – CBZ initial peak area.

Table S1. CBZ degradation efficiencies vs. photocatalyst dose and irradiation time, $[CBZ]_0$ = 8.75 mg/L = 3.71 × 10^{-5} M, photocatalyst dose = 100–800 mg/L, irradiation time = 15–45 min.

	15 min		30 min		45 min	
$[TiO_2]$, mg/L	[CBZ], mg/L	Efficiency, %	[CBZ], mg/L	Efficiency, %	[CBZ], mg/L	Efficiency, %
100	1.50	82.86	0.56	93.60	0.11	98.74
200	1.02	88.34	0.26	97.03	0.05	99.43
300	0.98	88.80	0.19	97.83	0.03	99.66
500	0.93	89.37	0.09	98.97	0.02	99.77
800	0.96	89.03	0.14	98.40	0.03	99.66

Method S1. CBZ determination method validation

1. Linearity

Stock solution was made in methanol, while the working solutions were performed in water. The MS detector response was tested between 0.01 and 10 mg/L carbamazepine and the method proved to be linear, with R^2 value of 0.9999.

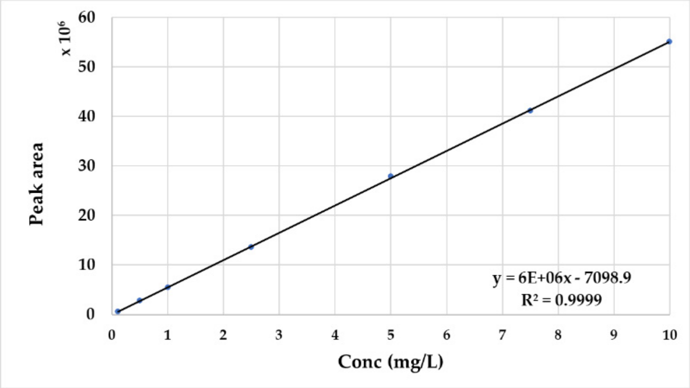

Method S1. - Figure 1. MS detector response

2. Sensibility

To test the selectivity of the method, a blank sample was analysed. No interferences have been observed on the retention time of carbamazepine. Thus, the developed method could be considered specific/selective.

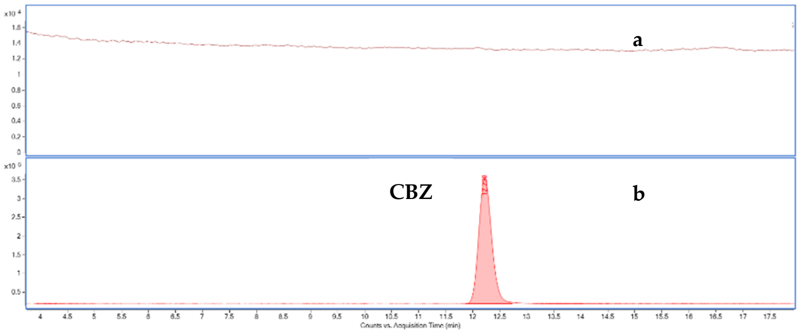

Method S1. - Figure 2. SCAN chromatogram of a blank sample that does not contain interferences (a) versus the SCAN chromatogram of a standard solution of 5 mg/L CBZ (b).

3. Precision

The accuracy of the method was determined by evaluating the inter-day and intra-day precision. The values were 4.2% and 7.5% for inter-day and intra-day experiments, respectively, which are very good considering the fact that the acceptability limit for an LC-MS method in up to 15%.

4. Sensitivity. Detection and quantification limit

Limit of quatification (LOQ) was determined as the lowest point of the calibration curve, such as 0.01 mg/L. The limit of detection was considered to be 2.8 time lower than the LOQ value, such as 0.0036 mg/L.

5. Uncertainty measurement

The uncertainity measurement was also calculated, resulting a value of only 17%, given the fact that no other sample preparation step was involved.

6. Analysis measurement

During the samples analysis, all experiments were performed in duplicate. A blank sample and a standard solution were used for each batch of samples. Values lower than LOQ were not involved in the kinetic evaluation.
